# Effects of the Application of a Program of Adapted Utilitarian Judo (JUA) on the Fear of Falling Syndrome (FOF) for the Health Sustainability of the Elderly Population

**DOI:** 10.3390/ijerph15112526

**Published:** 2018-11-12

**Authors:** Luis Toronjo-Hornillo, Carolina Castañeda-Vázquez, María del Carmen Campos-Mesa, Gloria González-Campos, Juan Corral-Pernía, Fátima Chacón-Borrego, Óscar DelCastillo-Andrés

**Affiliations:** Physical Education and Sports Department, University of Sevilla, 41013 Sevilla, Spain; ltoronjo@gmail.com (L.T.-H.); carolinacv@us.es (C.C.-V.); gloriagc@us.es (G.G.-C.); juancorral@us.es (J.C.-P.); fchacon@us.es (F.C.-B.); ocastillo@us.es (Ó.D.-A.)

**Keywords:** adults, falls, older adults, prevention, healthcare, injury

## Abstract

This research analyzes the fall history of a group of elderly people and studies the effects of an intervention program based on Adapted Utilitarian Judo (JUA) to teach fall control in subjects with fear of falling syndrome (FOF). We adopted a quasi-experimental research design with pre-post measurement of the experimental group, in a healthy, pre-fragile sample of 12 women aged 71.5 ± 8 years, chosen using non-probabilistic-incidental accessibility sampling. The WHO questionnaire was used for the functional assessment of the fall. To evaluate FOF, we applied the 16-item version of the Falls Efficacy Scale-International (FES-I), (pretest and posttest). This intervention program was based on Adapted Utilitarian Judo and conducted over 8 weeks, with two 60-minute sessions each week. After analyzing the scores obtained by the subjects in the pre and post FES-I, we found that the intervention with the JUA program had been significant for the experimental group with *p* ≤ 0.004, and there was an 11.9% decrease in the fear of falling (FES-I pos = 18.17). The results show that after the application of the JUA program there were significant improvements in subjects’ perception of FOF, with this being greater in those who had the highest levels of fear of falling before the intervention.

## 1. Introduction

A fall is an involuntary event that causes you to lose your balance, with your body hitting the ground or any other firm surface that stops it [1]. The World Health Organization [1] determines falls as the second major cause of accidental injuries leading to death and considers research and development programs related to them a priority. In addition, data on the self-reported consequences of falls in older adults show a significant number of injuries related to falls and a high cost of medical care [2]. 

Often linked to the risk of falling, appears the fear or dread of falling. Fear of Falling (FOF) is a set of signs and symptoms presented by a person with this problem. As a result, there may be a change in attitude and/or behavior that increases the risk of falling, injury, social isolation, deficits in self-care, anxiety, lack of recreational activities or impaired mobility, among others.

Tinetti, Richmond and Powell defined fear of falling (FOF) as the loss of confidence in oneself to avoid a fall when doing daily activities [3]. In the study of Fear of Falling Syndrome in people over 65 [4], this loss of confidence may range from slight concern about falling to refusing to do most daily tasks. 

To describe this syndrome, authors began to define it as a psychological consequence or trauma of the fall that led to a decrease in activities and a subsequent loss in the functional capacity of the elderly person. Fear of falling increases with age, the onset of illness, and in those older adults who have fallen in the previous year [5].

Initially, FOF was measured with a single dichotomous question based on a yes/no answer about their fear of falling. This is a simple method but produces good results and is useful when assessing fear of falling in the elderly [6]. Despite this, Tinetti and colleagues [3] consider that this question does not study the psychological impact of falls on older people comprehensively. Hence, on the basis of these works [3,7,8] from the theory of self-confidence, they developed the Falls Efficacy Scale (FES) which considers the fear of falling to be a decrease in the confidence of the elderly to perform daily activities without falling. Over this scale, some authors [9] found that the FES may discriminate between elderly adults who are afraid and those who are unafraid of falling (afraid M = 32.4; M unafraid = 19.7, *t* = 2.88, *p* < 0.001) and between those who avoid activities and those who do not avoid daily activities (avoid M = 43.4; do not avoid M = 19.9, *t* = 5.46, *p* < 0.001). 

To identify the variables that lead to this loss of confidence in the elderly to perform daily activities without falling, other studies have observed moderate correlations between FES scores and balance (*r* = 0.37 a 0.61); other studies have found that FES can predict whether subjects are likely to fall and decreased functional ability in the individual [10]. Along the same lines, recent work carried out on healthy adults over 60 has attempted to reduce fall rate by working on strength and/or balance with different physical activity (PA) programs, such as the use of slackline training [11,12], functional movement circles [13] or core instability strength training [14], amongst others. Other studies have used training adapted from judo (in some cases combined with pharmacological treatment) to improve bone mineral density (BMD) in postmenopausal women, to prevent and treat osteoporosis, balance and quality of life, with encouraging results [15,16]. An ecological model including individual and environmental factors should be considered when conducting research and designing programs and decision policies related to FOF for older adults with and without a history of falling [17].

In the last decade, more research has been done on landing strategies to reduce the impact of falls. The effects of an adequate fall technique reduce the risk of related injuries which are associated to multiple factors, such as location of the impact, the direction, and the magnitude of the loads applied to the body on impact [18,19].

In short, the review of the specialized literature shows that the main lines of action focus on fall prevention [20,21,22], while recognizing that older adults will always be susceptible to falling. As a result, we have conducted a program of Adapted Utilitarian Judo, which is a *significant comprehensive physical activity which leads participants to improve their physical condition, increasing balance capacity, functional gait, coordination and memory; introducing elements of generational and intergenerational socialization, all resulting in the increase of self-esteem, security, personal autonomy and therefore quality of life of the participants* [23] (p. 194). The objective was to improve the physical condition and health of older adults and reduce or minimize the risk of falls and their consequences, teaching older adults strategies for falling by reducing the impact of falling. We must not forget that, when working with a population of older adults in a sensitive area such as the realization of a “global program of training in falls”, a key element to take into account is the professional training of teachers. From the point of view of the researchers it should be oriented to the field of Physical Education and Health (Graduated in Physical Activity and Sports Sciences, Graduated in Physiotherapy, Specialized Master, Higher Technician in Physical Activity and Sports). This academic training can be reinforced, with experience in field work with the target population, and with specific training on the work of safe and protected falls.

This study investigated the fall history of a group of elderly people and analyzed the effects of applying an Adapted Utilitarian Judo program to teach fall control on FOF Fear of Falling Syndrome (STAC).

## 2. Materials and Methods 

### 2.1. Participants

Twelve women aged between 57 and 83 took part in this study (mean age of 71.5 ± 8 years). The anthropometric characteristics are adjusted to a weight of between 47.3 and 93.7 (average weight of 74.22 ± 14.61 kg), and a height of between 1.42 and 1.65 (average height of 1.54 ± 0.69 mts). They were selected using non-probabilistic-incidental sampling [24] for their accessibility (convenience sampling). The sample was classified as healthy and pre-fragile [25] within the fragility parameters for the elderly population. The design was quasi-experimental, with pre- and post- measures of the experimental group. 

As inclusion criteria, subjects had to be 55 or over and with no diagnosed illnesses that would prevent them from exercising. Subjects were excluded if, for medical reasons, they had been advised against taking physical exercise, had suffered from congestive heart failure, felt chest pain, dizziness or angina during exercise, or had uncontrolled high blood pressure (160/100).

Regarding the fall history of the sample (Table 1), 73% reported having suffered a fall at some point, 13% said they had changed their lifestyle as a result of a fall and 50% said they were afraid of falling again. 

**Figure 1:**
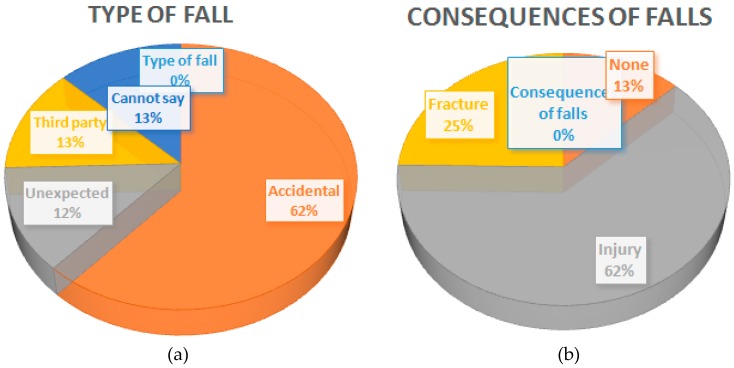
Type and consequences of falls. (**a**) Type of fall; (**b**) Consequences of falls.

As for the type and consequences of the falls (Figure 1), 62% said the fall was accidental, and the consequences in most cases were injuries (62%), followed by fractures (25%).

### 2.2. Instruments

For the functional assessment of the fall, we used the WHO questionnaire [26] for the study of falls in the elderly. To assess the STAC, we applied the 16-item version of the Falls Efficacy Scale-International (FES-I), before (pre-test) and after (post-test) the intervention. This scale analyzes confidence and skill at avoiding a fall while performing routine daily activities [27].

### 2.3. Procedure

We applied an intervention program based on Adapted Utilitarian Judo (JUA) **[28,29]** over the course of 8 weeks, with 2 weekly sessions each lasting 60 min. Some contents of a traditional Judo class have been adapted, contextualizing them to a utilitarian work directed to the specific requirements of the Older Adults, and they have been oriented to give a global treatment to the prevention of the falls and their consequences. The proposed exercises have been introduced following a progression of increasing difficulty, which has been graded according to the achievements and advances made by the participants. The repetitions of movements, already known, as well as the introduction of new movements, have been carried out, maintaining the principles of progression, assimilation, and above all, putting the safety of the participants in the program first. All subjects in the sample were informed about the objectives of the study and agreed to participate after giving informed consent.

Likewise, the program complies with the proposals of the Ethics Committee of the Biomedical Research of Andalusia, focused on ensuring the safety of the Older Adult throughout the execution process.

JUA is based on the technical contents of traditional Judo, or basics of Kodokan Judo, started in Japan in 1882 by the master Jigoro Kano. The characteristics of Judo techniques [30] and the reduction of the magnitude of impacts on the body by applying these techniques [30,31], are the most interesting factors for reducing the risk of injury in elderly people. 

With the focus on the different types of *ukemis* [32], we worked collaboratively on *yoko-ukemi* (lateral falls) and *ushiro-ukemi* (back falls) to enable the older person to develop and assimilate an effective, non-injurious technique for falling and rising from the ground. Our methodological proposal [33] concentrated on sport designs and social integration in which sports activities are tailored to meet the characteristics of the recipients.

To teach *ukemis* to older adults, we proposed activities of assimilation and assisted assimilation [34], linked to the necessary safety elements to prevent injuries, to enable them to automate technical moves in the most effective way. This learning process taught the elderly subjects to react reflexively in the event of a fall.

For the intervention with the elderly group, we proposed two learning sequences: one autonomous implement-assisted; and the other collaborative partner-assisted (with or without implements). In these exercises, we laid the emphasis on the social and entertaining side of what we were doing.

### 2.4. Data Analysis

We conducted a descriptive analysis using the SPSS (V.24.0, IBM, Armonk, NY, USA) statistical program, with the category variables presented in terms of frequencies and percentages, with the mean and standard deviation presented for the numerical variables. We used the *t*-test for paired samples to analyze significance in mean difference as it was a small sample size, we considered that the differences were significant when *p* ≤ 0.05.

## 3. Results

To analyze the evolution of scores for each subject, we took into account the cut-off points of the fear of falling using the 16-item version of the FES-I questionnaire [35]. We established a three-level classification: Ranging from low (16 to 19 points), to moderate (20 to 27 points) and high (28 to 64 points) fear of falling.

Of all the variables analyzed using the FES-I, Table 2 below shows that the JUA program significantly reduced the fear of subjects in the following situations: walking on a slippery surface, reaching up or down for something, walking in a crowded place, and going up and down a ramp. These are four of the five variables that received the highest score in the pretest (over 2 points on average), which means that they produced the most fear amongst the subjects. 

The JUA intervention program was therefore significant for the experimental group as Table 2 shows (*p* ≤ 0.004).

**Figure 2:**
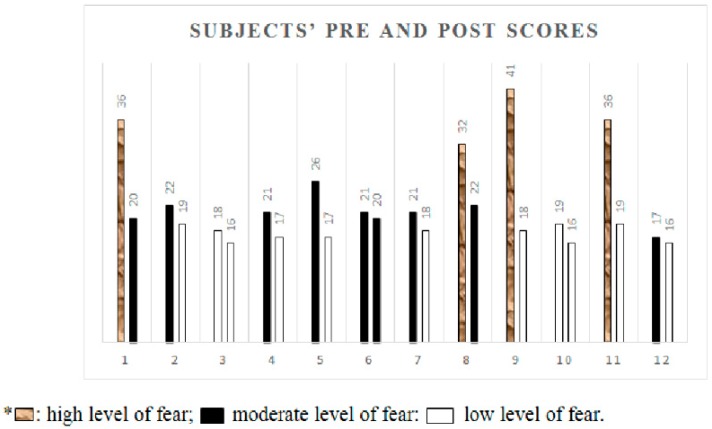
Scores obtained in the FES-I before and after the intervention according to levels of fear of falling.

**Figure 3:**
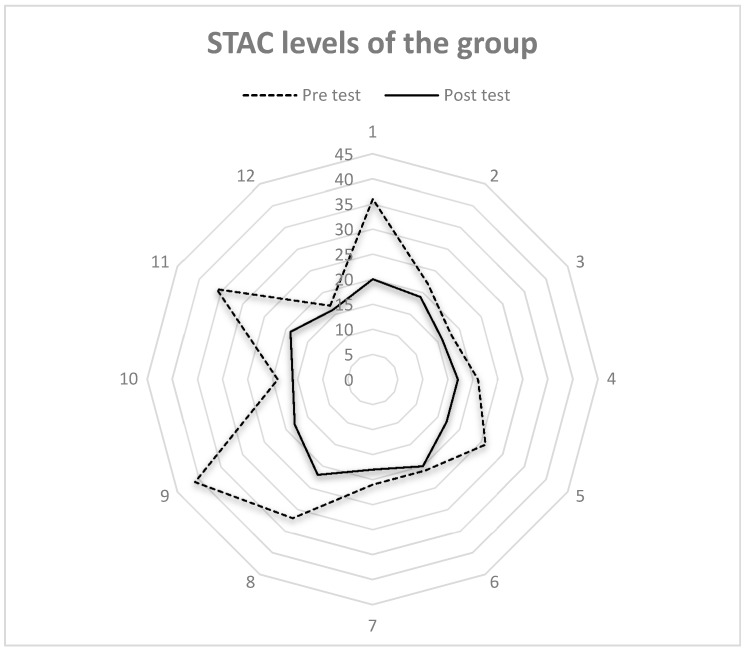
Spiderweb of experimental group subjects’ pre- and post- scores obtained in the FES-I.

Figure 2 and Figure 3 show the final scores obtained in the FES-I by each of the subjects in the experimental group, before and after the JUA intervention program. They show that after the intervention, all the subjects, bar two (1 and 8), reached a low level of fear of falling. Subjects 9 and 11 even managed to reduce their fear of falling by two levels, from high to low. Subjects 1 and 8 started from a high level of fear of falling and managed to reduce this with the program to a moderate level.

If we analyze the results at group level, as shown in Figure 3, after the intervention with the JUA program all subjects were located below the blue area, which means a low level of fear of falling (≤22 points) [35].

The personal Body Mass Index data (BMI) has been correlated through the Pearson correlation coefficient, with the results obtained in the measurements made on the fear of falling syndrome (STAC), and no significant statistical differences were found for this variable (pre-test *r* = 0.962 y con el post-test *r* = 0.844).

## 4. Discussion

The main findings of this study are substantial, since the results show that there were significant improvements in individuals’ perception of fear of falling, with these being greater in those subjects who had the highest levels of fear of falling before starting the intervention. As a result, some of the older people who participated in the study were able to return to daily activities that had become difficult for them because of the fear of falling.

The results show that the JUA program reduced the fear of falling by 11.9%, dropping from 40.35% before the intervention to 28.39% after participation in the program. As a consequence, we can state that this program substantially reduced the risk of suffering fall episodes, as the FES-I was 18.17 following the intervention, and an FES-I > 26 is a potent predictor of falls [29].

The results obtained in our study coincide with others proposing proprioceptive response strategies, applying therapies such as Tai-Chi that have obtained 11% reductions in the fear of falling, using the Activities-Specific Balance Confidence Scale questionnaire [30].

In most of these studies, fear of falling was reduced by improvement in dynamic balance [28,31]; however, none of the subjects with the worst level of fear managed to reduce their FES-I index below 23 points following treatment. In contrast, after our intervention we obtained results below 23 points in the twelve subjects, which leads us to recommend the application of this program together with work on dynamic balance.

There is a direct relationship between the practice of physical activity by older people and the fear of falling [32]. We would like to emphasize that as the fear of falling decreased, subjects began to see themselves as being capable of performing physical or daily activities such as Walking in a crowded place. This was one of the FES-I variables that received the highest score in our pretest (above 2 points on average) but which decreased considerably in the posttest (M = 1.58). 

Therefore, it is possible to teach fall control to older adults using the JUA program. Hence, there would seem to be strong reasons for including this program in the contents of structured physical activity for older people, along with work on other qualities, such as strength, which are important for the elderly [33].

It is important to remember that social and recreational programs are more likely to increase the quality of life of subjects [34]. The JUA program works in a social and entertaining way, promoting social interactions between older people. This made it possible for us to meet our objectives, as the JUA helped improve the quality of life of these people.

As the JUA program is a pioneering protocol for the reduction of FOF in older adults, and due to the characteristics of this protocol, it is difficult to compare this program with others found in the literature to discuss its limitations. We can point out as limitations of our studio the small size of the experimental sample was in part due to the exclusion criteria, since in this type of population we find frequent health problems. We did not gather information about the informal activity of the subjects or make any other measurements such as verifying the perceived quality of life or balance, amongst others. Another perspective to keep in mind in the future would be to collect other data taking into account other variables before and after starting the program such as, for example, carrying out a preliminary evaluation of the strength of arms and legs, of all the subjects participating in the program. Likewise, we adopted a quasi-experimental design, with no control group, so it would be desirable in order to enrich the results of new research, to count on the design with the presence of a Control Group.

## 5. Conclusions

In conclusion, the results obtained show that the application of a teaching program for the control of falls using JUA in older adults can help reduce FOF and therefore improve the quality of life of these subjects.

Looking ahead, there would seem to be a strong argument for including JUA with its innovative, social and entertaining approach, in physical activity programs promoting active ageing and fragility prevention in the elderly. In the light of the initial data obtained in this study, the program appears to provide an effective tool for working on control, and STAC reduction, for care and healthcare professionals working with the elderly.

## Figures and Tables

**Table 1:** Description of the sample according to fall history and incidents.

%	Have Suffered a Fall	First Fall	Have Fallen in Previous 6 Months	Influence of Fall on Lifestyle	Fear of Falling
Yes	73	56	0	13	50
No	27	44	100	87	50

**Table 2:** FEES-I pre-post intervention scores and values by variables analyzed and complete group.

Variables	Pretest		Post-Test		*t* Paired-Samples	
	M	SD	M	SD	*t*	Bilateral Sig.
Clean the house	1.43	0.79	1.00	0.00	1.82	0.096
Dress or undress	1.00	0.00	1.00	0.00	-	-
Prepare food	1.00	0.00	1.00	0.00	-	-
Bath or shower	1.33	0.88	1.00	0.00	1.30	0.22
Do the shopping	1.33	0.77	1.00	0.00	1.48	0.166
Sit down or get up from a chair	1.25	0.86	1.00	0.00	1.00	0.339
Go up or downstairs	1.83	1.03	1.42	0.51	1.82	0.096
Walk in the neighborhood (outside)	1.50	0.79	1.00	0.00	2.17	0.053
Reach up or down for something	2.08	0.90	1.17	0.57	3.52	0.005
Answer the phone	1.33	0.65	1.00	0.00	1.77	0.104
Walk on a slippery surface	2.83	0.93	1.67	0.65	4.31	0.001
Visit a friend or relative	1.42	0.79	1.00	0.00	1.82	0.096
Walk in a crowded place	2.17	1.11	1.17	0.38	3.31	0.007
Walk on an uneven surface	2.33	1.30	1.58	0.66	1.91	0.082
Go up and down a ramp	2.00	0.95	1.17	0.38	2.59	0.025
Go out for a social event	1.00	0.00	1.00	0.00	-	-
Pretest–Postest	25.83	8.23	18.17	1.89	3.60	0.004
